# Developmental Exposure to Atrazine Impairs Spatial Memory and Downregulates the Hippocampal D1 Dopamine Receptor and cAMP-Dependent Signaling Pathway in Rats

**DOI:** 10.3390/ijms19082241

**Published:** 2018-07-31

**Authors:** Jianan Li, Xueting Li, Haoran Bi, Kun Ma, Baixiang Li

**Affiliations:** 1 Department of Toxicology, College of Public Health, Harbin Medical University, Harbin 150081, China; lijianan512133@163.com (J.L.); lxt0451@163.com (X.L.); kris-mamm@sina.com (K.M.); 2 Department of Epidemiology, College of Public Health, Harbin Medical University, Harbin 150081, China; bihaoran1989@sina.com

**Keywords:** atrazine, learning and memory, hippocampus, D1 dopamine receptor

## Abstract

Atrazine (ATR) is a widely used herbicide that has been implicated as a neurotoxicant. Recent experimental evidence has implicated that ATR exposure also appears to have adverse effects on the hippocampus, which is a critical region for learning and memory. The aim of the present study was to investigate the effects of ATR toxicity on the hippocampus of developing rats. Postnatal day (PND) 28 male Sprague–Dawley (SD) rats received ATR by oral gavage at 10 or 100 mg/kg bodyweight (BW) for 30 consecutive days and were sacrificed at PND 90. Behavioral test results indicated that spatial learning and memory were affected by ATR treatment. Electron microscopy analysis showed that the ultrastructures of the hippocampus were altered in the ATR-treated groups, as compared to the control group. Additionally, ATR treatment impacted dopamine and D1 dopamine receptor (D1DR) contents through different mechanisms. Reduced mRNA and protein expression levels of factors involved in the cAMP-dependent signaling pathway were also detected. These results indicate that the developmental exposure of rats to ATR can damage the hippocampus and spatial memory, which might be related to the downregulation of expression levels of the D1DR and its downstream signaling pathway.

## 1. Introduction

Atrazine (6-chloro-*N*-ethyl-*N*′-(1-methylethyl)-1,3,5-triazine-2,4-diamine, ATR) is a broadly used herbicide worldwide. However, because of high levels in drinking water, the application of ATR is banned in the European Union and is highly restricted in the United States. Currently, the adverse effects of ATR exposure to humans remain unclear. Humans could be exposed to ATR during production or application in farming and forestry, while non-occupational exposure routes include ingestion, absorption, and inhalation [1].

Epidemiological studies indicate that the potential impact of ATR on humans primarily involves reproduction and development, but no causal association between ATR exposure and oncogenesis has yet been reported [2]. Experimental evidence obtained from animal studies demonstrated that ATR could be an endocrine disrupter, which influences hormone production and, subsequently, reproduction [3,4,5,6,7]. As hormones play a major role in the development of the central nervous system (CNS), recent research has suggested that ATR is a dopaminergic system toxicant. Exposure to 10 mg/kg bodyweight (BW) of ATR for 1 year was found to decrease dopamine concentrations in the striatum of male Sprague–Dawley (SD) rats [8], similar to that obtained in Albino rats intraperitoneally injected with a higher dose of ATR at 100 mg/kg BW [9]. In contrast, an increase in striatal dopamine and dopamine turnover were detected after acute oral exposure of C57BL/6 mice to ATR at 125 mg/kg BW [10]. The results of our previous study found that exposure to ATR at 100 mg/kg BW for 3 months induced autophagy and apoptosis-related changes in the dopaminergic neurons of male Wistar rats [11]. Dopamine modulates several functions of the CNS, including motivational processes [12], motor activity [13], learning, and memory [14]. The hippocampus is a key region in learning and memory formation, which could also be affected by ATR. However, the few studies that have examined the effects of ATR exposure on hippocampus-dependent learning and memory have reported mixed results. For example, Walters et al. found that exposure of Sprague–Dawley rats to ATR at 100 µg/kg or 10 mg/kg BW did not impair performance in the spatial discrimination reversal task or the Morris water maze (MWM) test [15], while Lin et al. revealed that ATR administered at >25 mg/kg BW for 10 days impaired performance of C57BL/6 mice in a novel object recognition task [10]. The same effect was also found even at a low concentration of ATR (3 mg/L) in dams and adult female C57BL/6 mice [16]. Moreover, exposure of male Wistar rats to ATR at 300 mg/kg BW for 7 days decreased performance in the Y-maze spontaneous alternation test [17].

Based on these inconsistent results, it is necessary to determine whether ATR impairs hippocampus-dependent learning and memory, and to identify the potential underlying mechanisms. Previous studies have suggested that dopamine may have a regulatory function in hippocampus-dependent synaptic plasticity and memory [18,19]. Dopamine modulates these effects through two types of G-protein-coupled receptors: the D1- and D2-like receptor, respectively [20]. A large number of studies demonstrated that the D1 dopamine receptor D1DR in the hippocampus is critical for learning and memory [21,22,23]. Dopamine receptors affect neuronal function mainly via regulation of the cyclic adenosine monophosphate (cAMP)-dependent signaling pathway, which includes activation of the protein kinase A (PKA) as well as phosphorylation in the cAMP response element-binding protein (CREB) [24]. Subsequently, phosphorylated CREB (p-CREB) modulates the expression of many proteins and immediate early genes (IEGs) that participate in the regulation of the learning and memory processes, such as brain-derived neurotrophic factor (BDNF), c-Fos and Zif268 [25].

The aims of the present study were to evaluate the detrimental effects of ATR exposure on the hippocampus of Sprague–Dawley rats using the MWM test and histopathological examinations, and to compare mRNA and protein expression levels of D1DR and the cAMP-PKA-CREB signaling pathway in the hippocampus. The results of this study are expected to expand current knowledge on the effects of ATR neurotoxicity on hippocampus-dependent learning and memory capabilities.

## 2. Results

### 2.1. Changes in Bodyweight (BW) and Physical Status

No animal died and there were no significant treatment-related differences in the BW and physical status of rats during the experimental period, as reported previously [8,10,11].

### 2.2. Effects of Atrazine (ATR) Exposure on Spatial Learning and Memory

**Figure 1:**
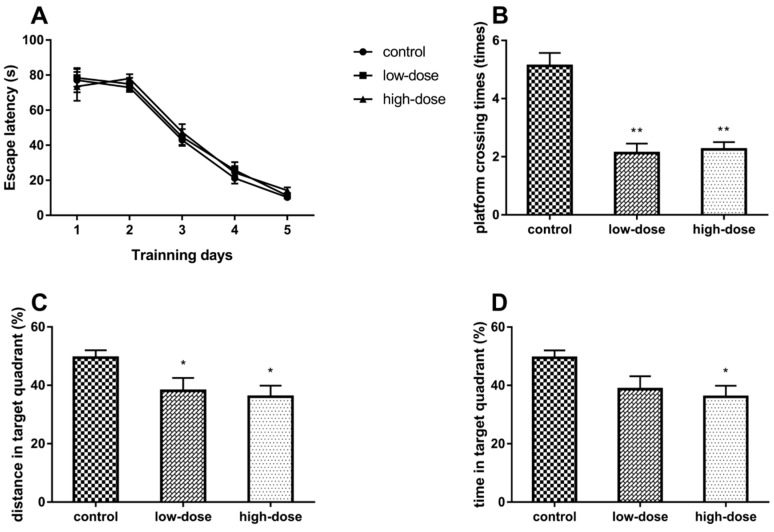
(**A**) Escape latency in the Morris water maze test on different training days; (**B**) Times of animal passing the site where there had been a platform; (**C**) Percentage of distance in the target quadrant; (**D**) Percentage of time that the rat spent in the target quadrant. Each column represents the mean ± standard error of mean (S.E.M), eight rats per group, * *p* < 0.05 vs. the control group, ** *p* < 0.01 vs. the control group.

The escape latency in the training phase had decreased in all groups. However, there was no significant difference in the time to reach the platform among all groups. (Figure 1A, analysis of variance (ANOVA): group effect: *F*_(2, 21)_ = 0.43, *p* = 0.66; time effect: *F*_(4, 84)_ = 129.34, *p* < 0.01; interaction effect between group and effect: *F*_(8, 84)_ = 0.23, *p* = 0.97).

The results of the spatial probe test phase indicated that ATR exposure significantly decreased the platform crossing times (Figure 1B, ANOVA: *F*_(2, 21)_ = 23.28, *p* < 0.01; control vs. low-dose: *p* < 0.01, control vs. high-dose: *p* < 0.01) and the percentage of distance in the target quadrant (Figure 1C, ANOVA: *F*_(2, 21)_ = 4.45, *p* = 0.02; control vs. low-dose: *p* = 0.04, control vs. high-dose: *p* = 0.04). In the high-dose group, the percentage of time in the target quadrant was significantly less than that of the control group (Figure 1D, ANOVA: *F*_(2, 21)_ = 3.81, *p* = 0.04; control vs. high-dose: *p* = 0.04). These results suggest that ATR could influence the spatial memory capacities of rats. 

### 2.3. Effects of ATR Exposure on the Ultrastructural Features of Hippocampal Neurons

**Figure 2:**
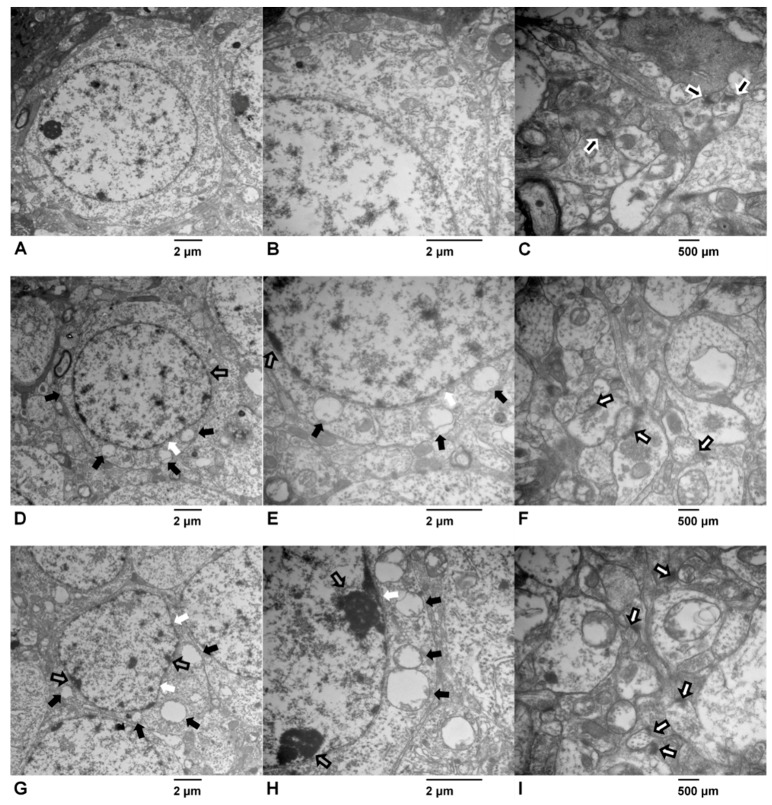
Effects of atrazine (ATR) on the ultrastructural features of the hippocampal neurons via transmission electron microscopy: control group: (**A**) 10,000×, (**B**) 20,000×; low-dose group: (**D**) 10,000×, (**E**) 20,000×; high-dose group: (**G**) 10,000×, (**H**) 20,000×. Effects of ATR exposure on the ultrastructural features of the hippocampal synapses via transmission electron microscopy: control group: (**C**) 30,000×; low-dose group: (**F**) 30,000×; high-dose group: (**I**) 30,000×. Six rats per group. Obvious changes are marked with different arrows, white arrows: fuzzy karyolemma; black arrows: swollen mitochondria; gray arrows with a black edge: concentrated chromatin granules; black arrows with a white edge: normal synaptic clefts and white arrows with a black edge: unclear synaptic clefts.

The results of transmission electron microscopy showed that the features of the hippocampal neurons of the experimental rats had changed in response to ATR exposure (Figure 2). The neurons in the control group had clear nuclei with smooth karyolemma bilayers, chromatin hypodispersion, and replete cytoplasm (Figure 2A). In addition, there was obvious infiltration of cytoplasm into the mitochondria, Golgi apparatus, and endoplasmic reticula (Figure 2B) with an abundance of synaptic vesicles in the presynaptic membrane and clear synaptic clefts (Figure 2C). In the ATR-treated groups, the karyolemma were fuzzy and shrunken, and this alternation was more prominent in the high-dose group (Figure 2D,G). The mitochondria were swollen with vacuolar degeneration and chromatin granules were condensed within the nuclear membrane (Figure 2E,H). In the ATR-treated groups, the only remarkable change in the synapses was that the synaptic clefts were unclear (Figure 2F,I).

### 2.4. Effects of ATR Exposure on Dopamine and cAMP Levels

**Figure 3:**
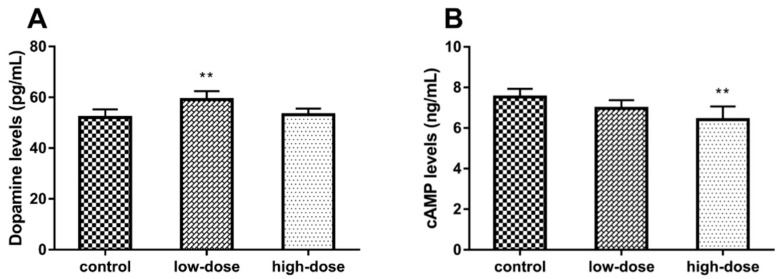
Effects of atrazine ATR exposure on dopamine and cAMP levels, as measured with an enzyme-linked immunosorbent assay. (**A**) Hippocampal dopamine levels in the control, low-dose, and high-dose groups (52.125 ± 3.386, 59.083 ± 3.585, and 53.151 ± 2.613 pg/mL, respectively). (**B**) Hippocampal cAMP levels in the control, low-dose, and high-dose groups (7.532 ± 0.436, 6.974 ± 0.439, and 6.410 ± 0.712 ng/mL, respectively). Each column represents the mean ± S.E.M, six rats per group, ** *p* < 0.01 vs. the control group.

The level of hippocampal dopamine in the low-dose group was significantly higher than that of the control and high-dose groups (Figure 3A, ANOVA: *F*_(2, 15)_ = 8.21, *p* < 0.01; control vs. low-dose: *p* < 0.01), while there was no significant difference between the control and high-dose groups (control vs. high-dose: *p* = 0.22). Hippocampal cAMP levels were significantly lower in the high-dose group as compared to the control and low-dose groups (Figure 3B, ANOVA: *F*_(2, 15)_ = 7.64, *p* < 0.01; control vs. high-dose: *p* < 0.01), while there was no significant difference between the control and low-dose groups (control vs. low-dose: *p* = 0.16).

### 2.5. Effects of ATR Exposure on D1 Dopamine Receptor (D1DR)-Positive Neurons in the Hippocampus

**Figure 4:**
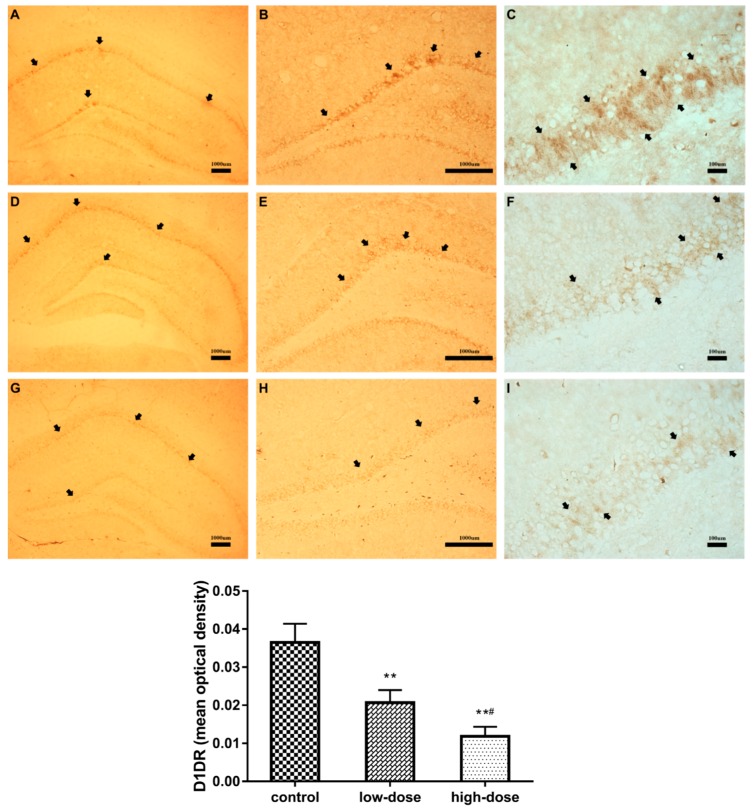
Effects of atrazine ATR exposure on D1 dopamine receptor (D1DR) positive neurons in the hippocampus, as determined by immunohistochemical analysis: control group: (**A**) 5×, (**B**) 20×, (**C**) 40×; low-dose group: (**D**) 5×, (**E**) 20×, (**F**) 40×; high-dose group: (**G**) 5×, (**H**) 20×, (**I**) 40×. Obvious changes are marked with black arrows. Optical densities were measured with Image-Pro Plus Image Processing Software. Each column represents the mean ± S.E.M, five rats per group, ** *p* < 0.01 vs. the control group. # *p* < 0.01 between ATR-treated groups.

The results of immunohistochemical analysis are shown in Figure 4. The trachychromatic D1DR-positive neurons in the control group were regular and densely arranged (Figure 4A–C). There was a significant dose-dependent decrease in the abundance of D1DR-positive neurons in the treatment groups (Figure 4, ANOVA: *F*_(2, 12)_ = 56.83, *p* < 0.01; control vs. low-dose: *p* < 0.01; control vs. high-dose: *p* < 0.01; low-dose vs. high-dose: *p* < 0.01). The D1DR-positive neurons in the low-dose group were regular and densely arranged, but staining was reduced, as compared to the control group (Figure 4D–F). Moreover, in the high-dose group, the D1DR-positive neurons were sparsely arranged with weaker staining as compared to the low-dose group (Figure 4G–I).

### 2.6. Effects of ATR Exposure on mRNA Expression Levels

**Figure 5:**
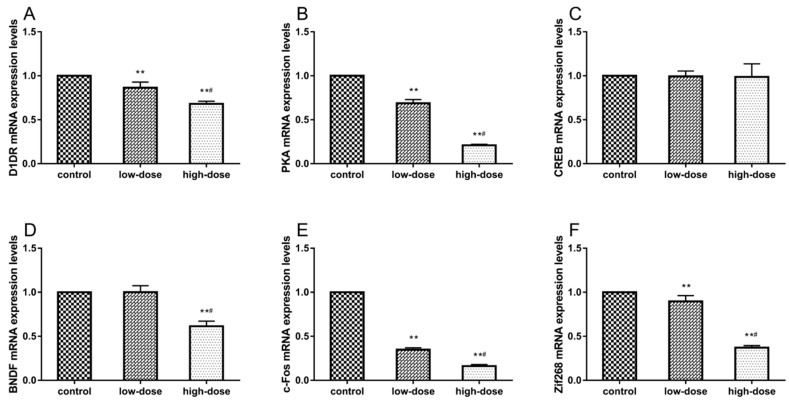
Effects of ATR exposure on mRNA expression levels in the hippocampus were measured with reverse transcription-PCR. (**A**) D1DR; (**B**) Protein kinase A (PKA); (**C**) cAMP response element-binding protein (CREB); (**D**) Brain-derived neurotrophic factor (BDNF); (**E**) c-Fos, and (**F**) Zif268. Expression levels were standardized to β-actin. Each column represents the mean ± S.E.M, six rats per group, ** *p* < 0.01 vs. the control group. # *p* < 0.01 between ATR-treated groups.

The mRNA expression levels of D1DR, PKA, CREB, BDNF, c-Fos, and Zif268 in the hippocampus are shown in Figure 5. There were significant decreases in mRNA levels of D1DR (Figure 5A, ANOVA: *F*_(2, 15)_ = 92.74, *p* < 0.01; control vs. low-dose: *p* < 0.01; control vs. high-dose: *p* < 0.01; low-dose vs. high-dose: *p* < 0.01) and PKA (Figure 5B, ANOVA: *F*_(2, 15)_ = 1566, *p* < 0.01; control vs. low-dose: *p* < 0.01; control vs. high-dose: *p* < 0.01; low-dose vs. high-dose: *p* < 0.01) in the ATR-treated groups, while there were no significant differences in CREB mRNA levels, as compared with the control group (Figure 5C, ANOVA: *F*_(2, 15)_ = 0.03, *p* = 0.97). ATR treatment significantly decreased the BDNF mRNA expression level in the high-dose group (Figure 5D, ANOVA: *F*_(2, 15)_ = 105.5, *p* < 0.01; control vs. high-dose: *p* < 0.01; low-dose vs. high-dose: *p* < 0.01). The c-Fos (Figure 5E, ANOVA: *F*_(2, 15)_ = 4744, *p* < 0.01; control vs. low-dose: *p* < 0.01; control vs. high-dose: *p* < 0.01; low-dose vs. high-dose: *p* < 0.01) and Zif268 (Figure 5F, ANOVA: *F*_(2, 15)_ = 434.2, *p* < 0.01; control vs. low-dose: *p* < 0.01; control vs. high-dose: *p* < 0.01; low-dose vs. high-dose: *p* < 0.01) mRNAs levels were significantly decreased in the ATR-treated groups.

### 2.7. Effects of ATR Exposure on Protein Expression Levels

**Figure 6:**
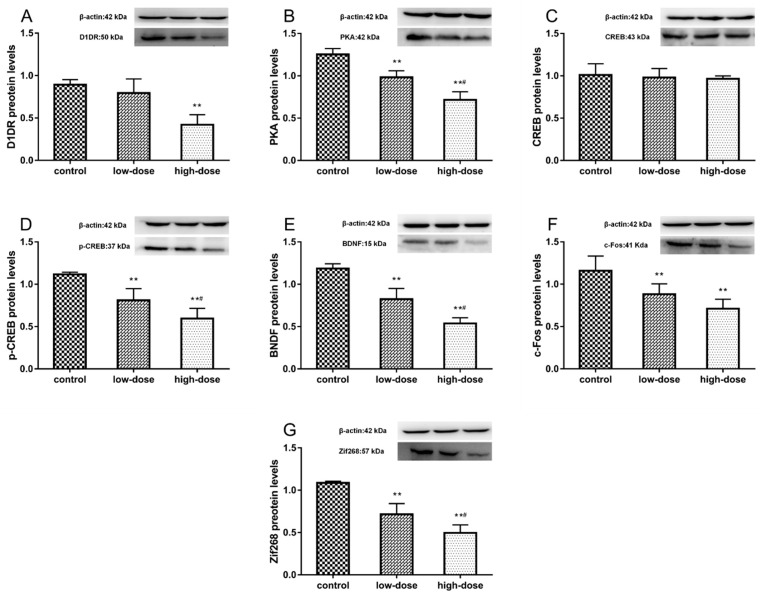
Effects of atrazine (ATR) exposure on protein expression levels in the hippocampus were measured using western blot analysis. (**A**) D1DR, (**B**) PKA, (**C**) CREB, (**D**) p-CREB, (**E**) BDNF, (**F**) c-Fos, and (**G**) Zif268. Expression levels were standardized to β-actin. Each column represents the mean ± S.E.M, six rats per group, ** *p* < 0.01 vs. the control group. # *p* < 0.01 between ATR-treated groups.

The protein expression levels of D1DR, PKA, CREB, p-CREB, BDNF, c-Fos, and Zif268 in the hippocampus are shown in Figure 6. ATR treatment significantly decreased the D1DR protein expression levels in the high-dose group (Figure 6A, ANOVA: *F*_(2, 15)_ = 24.76, *p* < 0.01; control vs. high-dose: *p* < 0.01). There was a significant decrease in PKA protein levels in the treatment groups (Figure 6B, ANOVA: *F*_(2, 15)_ = 66.06, *p* < 0.01; control vs. low-dose: *p* < 0.01; control vs. high-dose: *p* < 0.01; low-dose vs. high-dose: *p* < 0.01). There was no change in CREB protein levels in response to ATR treatment in all groups (Figure 6C, ANOVA: *F*_(2, 15)_ = 0.33, *p* = 0.72). The protein levels of p-CREB (Figure 6D, ANOVA: *F*_(2, 15)_ = 36.33, *p* < 0.01; control vs. low-dose: *p* < 0.01; control vs. high-dose: *p* < 0.01; low-dose vs. high-dose: *p* < 0.01) and BDNF (Figure 6E, ANOVA: *F*_(2, 15)_ = 80.61, *p* < 0.01; control vs. low-dose: *p* < 0.01; control vs. high-dose: *p* < 0.01; low-dose vs. high-dose: *p* < 0.01) were significantly decreased in the treatment groups. ATR treatment significantly decreased c-Fos protein expression levels in the treatment groups (Figure 6F, ANOVA: *F*_(2, 15)_ = 16.27, *p* < 0.01; control vs. low-dose: *p* < 0.01; control vs. high-dose: *p* < 0.01), but there were no significant differences between treatment groups (low-dose vs. high-dose: *p* = 0.11). There was a significant decrease in Zif268 protein levels in the treatment groups (Figure 6G, ANOVA: *F*_(2, 15)_ = 63.61, *p* < 0.01; control vs. low-dose: *p* < 0.01; control vs. high-dose: *p* < 0.01; low-dose vs. high-dose: *p* < 0.01).

## 3. Discussion

ATR has been among the most widely used artificial chemical pesticide worldwide over the last few decades. The widespread application of ATR and its persistent existence in the environment have continued to gain extensive attention. Numerous recent reports have highlighted the adverse effects of ATR exposure on the CNS [8,9,10,11]. The developmental phase is a key period of constructing brain function, including learning and memory [26,27]. Hence, the aim of this study is to identify effects of ATR exposure during the developmental phase on the learning and memory capabilities of rats. The major finding of the present study is that ATR has long-term deleterious effects on spatial memory and ultrastructure of the hippocampal neurons of rats, which may be related to the downregulation of D1DR and dysregulation of crucial factors in downstream signaling pathways.

The MWM test is commonly used to assess the hippocampal circuitry of rodents by evaluating spatial learning and memory capabilities [28]. The results of the present study showed that there were no significant differences in the escape latency between the control and experimental groups. However, in the spatial probe test, the platform crossing times, percentage of time, and distances covered in the target quadrant were lower in the ATR-treatment groups as compared to the control group. We hypothesized that ATR may not have an obvious effect on spatial learning, thus a new starting position was selected for use in the spatial probe test. The test results showed that spatial performance is a reflection of spatial memory, rather than a specific swim path [29], suggesting that ATR could impair the hippocampus-dependent spatial memory of rats. Notably, this result is inconsistent with that of a previously study reporting that ATR did not affect the performance of rats in the MWM test [15]. A possible explanation for this discrepancy is that the rats in this previous study had performed a spatial discrimination task for 3 months before the MWM test, and this behavioral training may be a type of environmental enrichment that could have affected performance in the MWM test [30].

The hippocampus is recognized as an important region of the brain for both acquisition and consolidation of memory [31]. In the present study, the results of transmission electron microscopy showed that the ultrastructure of neurons in the ATR-treated groups were strikingly altered with degeneration of mitochondria and nuclei, especially in the high-dose group, in agreement with the results of our previous study that the ultrastructure of striatal neurons was damaged by ATR [11]. However, few studies have investigated the histopathological effects of ATR exposure on the ultrastructure of the hippocampal neurons, and only one reported histopathological alternation to the hippocampus in response to ATR at 100 µg/kg BW, which included a significant reduction in the total number of neurons, perikaryal swelling, and astrocytic formations in the dentate gyrus of female mice [32]. Nonetheless, other pesticides, such as paraquat and parathion, are known to cause histopathological damage to the hippocampal neurons, including pyknosis, irregular arrangement, and apoptosis, which could lead to impairment of hippocampus-dependent learning and memory [33,34]. Another major finding of the electron microscopy analysis conducted in the present study was that the synaptic clefts in the ATR-treated groups were not as clear, as compared with the control group. The synapse is a critical structure of the neuron and synaptic connections that link neurons in the brain, which are considered to form the basis of memory [35]. Thus, we proposed that the blurred synaptic clefts may be a reason for the impairment of spatial memory in the ATR-treated groups.

Previous studies have demonstrated that ATR is a dopaminergic system toxicant, especially to dopamine in the striatum [8,9,10,15]. There is a strong relationship between dopamine content and hippocampal function, although the hippocampus is not a classic projective area of dopaminergic neurons. In this study, the amount of dopamine in the hippocampus was measured with an ELISA, which showed that the level of hippocampal dopamine in the low-dose group was significantly higher than that of both the control and high-dose groups. Dopamine is an important neurotransmitter in the brain and exerts its functions by activation of dopamine receptors [36]. D1DR is considered to play a dominant role in the long-term potentiation (LTP) of the hippocampus and memory formation [21,36]. In spatial learning and memory tasks, as in the MWM test, mice lacking D1DR exhibited severe defects [37,38]. Likewise, our results showed that the protein and mRNA expression levels of D1DR were significantly decreased in a dose-dependent manner in the ATR-treated groups. So, we hypothesized that the increased dopamine content in the low-dose group may have been a compensatory increase for the decrease in D1DR expression [39]. The cAMP-dependent signaling pathway is activated downstream of D1DR [24], which is an underlying mechanism of learning and memory, and may also, therefore, be affected by ATR.

Coupling of the D1DR with adenylyl cyclase causes an increase in cAMP production, which then activates the downstream cAMP-dependent signaling pathway and subsequent activation of PKA and CREB [40]. As expected, our results suggested that the cAMP levels in the high-dose group were significantly lower than in the control group. PKA is activated by cAMP in the cytoplasm and then transported into the nucleus, where it phosphorylates serine 133 of the CREB protein [41]. The p-CREB modulates IEGs expression and synaptic protein synthesis, which are thought to also participate in the regulation of learning and memory [25,42]. c-Fos and Zif268 are two critical IEGs in the LTP of the hippocampus, as c-Fos plays a key role in maintenance of the LTP and Zif268 regulates the duration of the LTP [43]. Besides, BDNF is a secretory protein that is crucial for neuronal survival and hippocampus-dependent memory formation [44,45,46]. Thus, based on the decrease of D1DR and cAMP by exposure to ATR, the members of the cAMP-dependent signaling pathway were selected to investigate the potentiation of ATR-induced impairment of spatial learning and memory. The results demonstrated that ATR exposure decreased the protein and mRNA expression levels of PKA, BDNF, c-Fos, and Zif268 in the hippocampus. However, the protein and mRNA expression levels of CREB were not affected by ATR treatment, although the protein levels of p-CREB were significantly decreased in the ATR-treated groups. We thus proposed that ATR exposure may only influence the phosphorylation of CREB in the hippocampus. These results are consistent with those of previous studies suggesting that the cAMP-dependent signaling pathway and its downstream molecules were affected by other hippocampal toxicants, such as amyloid-beta peptide, aluminum, and lipopolysaccharides [47,48,49,50]. Collectively, our findings suggest that developmental exposure to ATR impaired the spatial memory capabilities of rats, altered the ultrastructure of hippocampal neurons and downregulated the D1DR as well as the cAMP-PKA-CREB signaling pathway in the hippocampus.

## 4. Materials and Methods

### 4.1. Ethics Statement

The animal experiments in the current study were approved by the Medical Ethics Committee of Harbin Medical University (Harbin, China, HMUPHIRB2016006, 4 March 2016) and carried out in line with the Guidelines for the Care and Use Laboratory Animals established by the National Institutes of Health.

### 4.2. Animals and ATR Exposure

Seventy-five, 21-day-old, male, Sprague–Dawley rats were obtained from Beijing Vital River Laboratory Animal Technology Co., Ltd. (Beijing, China) and housed under a 12-h inverted dark/light cycle at a constant temperature of 18–22 °C and a relative humidity of 50%. After 1 week of acclimatization, the rats were randomly assigned to one of three groups of 25 rats each: a control group (1% methyl cellulose; Sigma–Aldrich Corporation, St. Louis, MO, USA); a low-dose group (10 mg/kg BW ATR, 98% pure; Trust Chem Co., Ltd., Shanghai, China), and a high-dose group (100 mg/kg BW ATR). The rats received oral gavage of ATR or methyl cellulose (controls) each day from Postnatal day (PND) 28 to PND 58, and this period is critical for cognitive capabilities construction [26,27]. ATR dosages were selected according to the reported median lethal oral dose and minimal damage dose of memory in rats [8]. BW was recorded weekly. On PND 90, the animals from each group were randomly divided into four parts for different experiments: 8 rats for behavioral testing; 5 rats for immunohistochemical analysis; 6 rats for transmission electron microscopy and enzyme-linked immunosorbent assay; 6 rats for reverse transcription-polymerase chain reaction analysis and western blot analysis. The hippocampal tissue collection was performed as described by our previous study [51]. 

### 4.3. Morris Water Maze (MWM) Test

The spatial learning and memory capabilities of the rats were evaluated with the MWM test. The apparatus consisted of a circular black water tank (180 cm in diameter and 58 cm deep) filled with water (about 42 cm deep) at a temperature of 19–21 °C. The apparatus was closed by black curtains, with extra visual cues inside the curtains. The water tank was divided into four equal quadrants: north-west, north-east, south-west, and south-east. A circular platform (10 cm in diameter) was located at ~2 cm below the water surface in the north-east quadrant. The test period was divided into two phases [29].

Phase 1 (training phase): The rats underwent four training sessions each day for 5 consecutive days. During each training session, the rats were placed into the water facing the tank wall in a set of semi-randomly selected distal starting positions each day. The escape latency was set to 90 s. The rats were kept on the platform for 10 s before removal. Rats that failed to find the platform within 90 s were picked up and placed on the platform for 10 s.

Phase 2 (spatial probe test): On day 6, the rats underwent a 90-s probe test with the platform removed from the tank. In brief, the rats were placed in a novel starting position. The platform crossing times, as well as the percentage of time and distance in the target quadrant were recorded over a 90-s period.

### 4.4. Transmission Electron Microscopy

After induction of deep anesthesia with pentobarbital sodium, the hippocampus was removed and cut into 1-mm^3^ pieces, which were fixed in 2% glutaraldehyde in 0.01 mol/L phosphate-buffered solution (PBS; Beijing Solarbio Science & Technology Co., Ltd., Beijing, China) for 48 h, then fixed again in a 1% osmium tetroxide for 2 h, dehydrated, and embedded in resin. Ultrathin sections were cut, stained with lead citrate, and assessed under a transmission electron microscope (JEM-2100; JEOL Ltd., Tokyo, Japan).

### 4.5. Enzyme-Linked Immunosorbent Assay (ELISA) for Dopamine and cAMP

The concentrations of dopamine and cAMP in the hippocampus were measured with commercial ELISA kits (Elabscience Biotechnology Co., Ltd., Wuhan, China), in accordance with the manufacturer’s instructions. In brief, the standard working solution and hippocampal tissue solution were added to the wells of micro ELISA plates and immediately mixed with biotinylated antibody (Ab) detection working solution. Then, the plate was covered and incubated at 37 °C for 45 min. After decanting the solution from each well, the plate was washed three times. Afterward, horseradish peroxidase (HRP)-conjugated working solution was added to each well and the plate was again covered and incubated at 37 °C for 30 min. The solution was then decanted from each well and the plate was washed five times. After the addition of substrate reagent, the plate was covered and incubated at 37 °C for 15 min. Stop solution was added to each well and the optical density of 450 nm was determined using a microplate reader (Bio-Tek Elx800; BioTek Instruments, Inc., Winooski, VT, USA).

### 4.6. Immunohistochemical Analysis

After induction of deep anesthesia, the animals were perfused through the aorta with PBS followed by chilled 4% paraformaldehyde in 0.01 M PBS (pH 7.2). The whole brains were removed and fixed in 4% paraformaldehyde at 4 °C for 48 h, then dehydrated in a gradient sucrose series in 0.01 M PBS until the organs sank. After dehydration, 10-mm coronal sections of the brain were cut using a cryostat (CM1900; Leica Microsystems GmbH, Wetzlar, Germany) and mounted onto poly-L-lysine-coated slides (Boster Biological Technology, Ltd., Wuhan, China). The sections were blocked with 2% goat serum in 0.01 M PBS for 40 min at room temperature, followed by incubation with a rabbit anti-D1DR polyclonal primary Ab in dilution fluid (dilution, 1:100; ImmunoWay Biotechnology Company, Plano, TX, USA) overnight at 4 °C. After washing six times in PBS, the sections were incubated with goat anti-rabbit immunoglobulin (Ig)G secondary Ab (ZSGB-BIO, Beijing, China) at 37 °C for 1 h, then washed again six times with PBS and incubated with ABC fluid (A:B:PBS = 1:1:100) at 37 °C for 30 min. The immunoreaction was visualized with the DAB kit (ZSGB-BIO) and terminated by washing with tap water. The sections were then air-dried and covered with neutral gum. Images were recorded using a fluorescence microscope (BX51; Olympus Corporation, Tokyo, Japan). The mean optical density of D1DR in the dentate gyrus region was analyzed using Image-Pro Plus Image Processing Software (v 6.0.0.260; Media Cybernetics, Inc., Rockville, MD, USA); briefly, the images were corrected with the optical density and the staining parameter was set; the mean optical density was determined by calculating the ratio of staining optical density to the staining area.

### 4.7. RNA Collection and Reverse Transcription-Polymerase Chain Reaction (RT-PCR) Analysis

Hippocampal total RNA was extracted with TRIzol Reagent and synthesized into cDNA according to the manufacturer’s instructions (Takara Bio, Inc., Shiga, Japan). The following primers were designed and synthesized to examine the mRNA expression levels of D1DR, PKA, CREB, BDNF, and Zif-268, (Generay Biotech, Co., Ltd., Shanghai, China): β-actin (forward: GAGAGGGAAATCGTGCGT; reverse: GGAGGAAGAGGATGCGG); D1DR (forward: TGTTTGTGTGGTTTGGGTGG; reverse: TATGGCATTATTCGTAGTAGGGC); PKA (forward: CAGAAGGTGGTGAAGCTGAAGCA; reverse: ACCTCCCAATCCGCCGTAAGT); CREB (forward: CCCCTGGAGTTGTTATGGCGT; reverse: ATTCTCTTGCTGCTTCCCTGTTCT); BDNF (forward: TCTACGAGACCAAGTGTAATCCCAT; reverse: GAAGTGTCTATCCTTATGAACCGC); c-Fos (forward: GGAGAATCCGAAGGGAAAGGAATAA; reverse: CGGTGGGCTGCCAAAATAAACT); and Zif-268 (forward: TTGTCTGCTTTCTTGTCCTTC; reverse: TTCAGTCGTAGTGACCACCTT). For data analysis, the relative mRNAs expression levels of the target genes were normalized to that of β-actin by the formula: Δ*C*t = (*C*t_target_ − *C*t_β-actin_); ΔΔ*C*T = (Ct_target_ − *C*t_β-actin_) treatment − (*C*t_target_ − *C*t_β-actin_) control. The 2^−ΔΔ*C*t^ method was used to calculate the relative expression levels of the target mRNAs. 

### 4.8. Western Blot Analysis

Total proteins of the hippocampal tissue samples were extracted with lysis buffer containing phenylmethane sulfonyl fluoride and a phosphatase inhibitor (Beyotime Institute of Biotechnology, Shanghai, China). The mixture was then incubated on ice for 2 h and centrifuged at 12,000× *g* for 10 min at 4 °C. Then, the supernatant was isolated and the protein concentration was measured using a commercial bicinchoninic acid protein assay kit (Beyotime Institute of Biotechnology). Total protein (80 mg) was separated with sodium dodecyl sulfate polyacrylamide gel electrophoresis (10%, 12% or 15%) and then transferred to a polyvinylidene fluoride membrane. The membranes were blocked with 1% bovine serum albumin or 5% non-fat milk in Tris-buffered saline/Tween 20 (TBST) for 1 h at room temperature and incubated at 4 °C overnight with an anti-D1DR polyclonal primary Ab (diluted 1:1000 in blocking buffer; ImmunoWay Biotechnology Company, Plano, TX, USA), anti-PKA polyclonal primary Ab (diluted 1:500 in blocking buffer; Millipore Corporation, Billerica, MA, USA), anti-CREB polyclonal primary Ab (diluted 1:1000 in blocking buffer; ImmunoWay Biotechnology Company), anti-p-CREB polyclonal primary Ab (diluted 1:1000 in blocking buffer; ImmunoWay Biotechnology Company), anti-BDNF polyclonal primary Ab (diluted 1:1500 in blocking buffer; Abcam Limited, Cambridge, UK), anti-c-Fos polyclonal primary Ab (diluted 1:1000 in blocking buffer; Abcam Limited), anti-Zif68 polyclonal primary Ab (diluted 1:1000 in blocking buffer; Abcam Limited), with anti-β-actin polyclonal Ab (diluted 1:1000 in blocking buffer; ImmunoWay Biotechnology Company) as the control. The next day, the membranes were washed six times with TBST and incubated with (HRP)-conjugated goat anti-rabbit IgG Ab as the secondary Ab (diluted 1:5000 in blocking buffer; ImmunoWay Biotechnology Company) for 1 h at room temperature. Then, the membranes were washed six times with TBST buffer and the immunoreactive bands were detected with enhanced chemiluminescent substrate included with a commercial western blot detection kit (Beyotime Institute of Biotechnology). The bands were recorded using a chemiluminescence system (Tanon-5200; Tanon Science & Technology Co., Ltd., Shanghai, China) and analyzed using ImageJ software (v 1.51; NIH., Bethesda, MD, USA).

### 4.9. Statistical Analyses

Results are presented as the mean ± SEM. Differences in escape latency, as measured with the MWM test, were identified by repeated-measures two-way analysis of variance (ANOVA). Other data were analyzed by one-way ANOVA. The Dunnett’s multiple comparison test was used for pairwise comparisons. Differences were considered statistically significant at * *p* < 0.05. All statistical analysis was performed using SPSS 18.0 software (SPSS, Inc., Chicago, IL, USA).

## 5. Conclusions

The results of the present study demonstrated that developmental exposure to ATR damaged the spatial memory capabilities of male Sprague–Dawley rats. The ultrastructures of the hippocampal neurons were also affected by ATR. In addition, the dopamine and D1DR levels in the hippocampus were influenced via different mechanisms, while the downregulation of the cAMP-dependent signaling pathway was also detected in the hippocampus after ATR treatment. However, in this study, it remains unclear whether the downregulation of D1DR and the cAMP-dependent signaling pathway is the underlying mechanism of ATR-induced impairment of spatial memory. Therefore, further investigations are warranted to validate this issue.

## Abbreviations

ATR	Atrazine
PND	Postnatal day
CNS	Central nervous system
D1DR	D1 dopamine receptor
cAMP	Cyclic adenosine monophosphate
PKA	Protein kinase A
CREB	cAMP response element-binding protein
BDNF	Brain derived neurotrophic factor
IEGs	Immediate early genes
MWM	Morris water maze
Elisa	Enzyme-linked immunosorbent assay
RT-PCR	Reverse transcription-polymerase Chain reaction
PBS	Phosphate buffered solution
TBST	Tris-buffered saline with Tween 20
